# Cardiac surgical interventions after coronary stent implantation

**DOI:** 10.1186/1749-8090-8-S1-O189

**Published:** 2013-09-11

**Authors:** B Matlakovics, M Vaszily, O Ignáczy, T Sugár

**Affiliations:** 1 Hungarian Defense Forces, Health Center, Department of Cardio-Thoracic and Vascular Surgery, Budapest, Hungary

## Background

At present times the frequency of stent implantation is increases. The anti-platelet therapy (APT) after stent implantation is necessary, but it could be responsible for increased postoperative bleeding, mortality and other complications.

## Methods

We collected 180 patients who previously underwent stent implantation, later on they underwent cardiac surgical intervention in our department. 151 CABG operation (50 off pump (OPCAB), 101 on pump), and 29 valve interventions where done. In the control group we had 2116 patients (1177 CABG, 939 valve). We detected the incidence of bleeding caused resternotomy, major cardiovascular events (repeat revascularization, myocardial infarction, stroke etc.), and the postoperative mortality.

## Results

The mortality was higher, when the patients had previously stent implanted (6,4% vs. 3,2% CABG, and 27,3% vs. 2,5% AVR). The number of bleeding complications, and the mortality was significantly higher in case of previously DES, compared to BMS implantations (mortality 33,3% vs. 23,8%, bleeding caused complications 22,2% vs. 4,8%). We did not find difference in the mortality between the on pump and OPCAB techniques, but the incidence of bleeding complications was higher in OPCAB cases (4% vs. 1,6%). Incidence of bleeding complications and the mortality were significantly higher in the early operations (within 42 days) compared to the late ones (mortality 10% vs. 3% and resternotomy 20% vs. 2,1%).

## Conclusion

It is not any difference in the postoperative outcome between on pump and OPCAB techniques, but the incidence of the bleeding complications is higher in case of the OPCAB operations. Based on this data, the on pump revascularisation technique is favorable after stent implantation. The postoperative mortality and incidence of the complications are higher in cases of early operations. Therefore the operative results are better in cases of late operations after stent implantation.

